# 727. Predictors of Depressive Symptoms in Pregnant Partners in Seroconcordant Couples Living with HIV in Zambézia Province, Mozambique

**DOI:** 10.1093/ofid/ofab466.924

**Published:** 2021-12-04

**Authors:** Daniel Sack, Ariano Matino, Graves Erin, Almiro Emilio, Bryan Shepherd, Caroline DeSchacht, Carolyn Audet

**Affiliations:** 1 Vanderbilt Medical Scientist Training Program, Nashville, TN; 2 Friends in Global Health, Quelimane, Zambezia, Mozambique; 3 Vanderbilt University, Nashville, TN; 4 Vanderbilt University Medical Center, Nashville, TN

## Abstract

**Background:**

Depression contributes to HIV treatment outcomes in sub-Saharan Africa, where approximately 15% of people living with HIV have comorbid depression. HoPS+, a cluster randomized trial among seroconcordant couples living with HIV, assesses male partner involvement during antenatal HIV care and HIV outcomes. We describe predictors of depressive symptoms among pregnant partners living with HIV in Zambézia Province, Mozambique.

**Methods:**

This baseline cross-sectional analysis includes 1079 female HoPS+ participants. We show demographic (age, enrollment date, relationship status, education, and occupation) and clinical (WHO HIV stage, body mass index [BMI], and antiretroviral therapy [ART] use history) factors. We model females’ depressive symptoms (Patient Health Questionnaire-9 [PHQ-9]) using proportional odds models with continuous covariates as restricted cubic splines (enrollment date, age, BMI, partner’s PHQ-9 score), categorical covariates (district, relationship status, education, occupation, WHO stage), and ART use history. Missing covariates were imputed 20 times.

**Results:**

Participants’ median age was 23 (interquartile range [IQR] 20-28). Most women reported no or < 7 years of education (84.1%), were farmers (61.3%), and were WHO stage I (81.9%). They had a median PHQ-9 score of 3 (IQR 0-5) and 47 (43.6%) had moderately severe or severe depressive symptoms, with 19.6% missing PHQ-9 scores. Among 867 pregnant partners with PHQ-9s, demographic and clinical covariates were not meaningful predictors of PHQ-9 score. Male partner’s PHQ-9 score, however, was associated with (covariate-adjusted Spearman’s rho 0.58, 95% Confidence Interval [CI]: 0.51-0.65) and strongly predictive of a pregnant partner’s score (Figure). An increase in a male partner’s PHQ-9 score from 9 to 10 was associated with 1.47 times increased odds (95% CI: 1.37-1.58) of a ≥1-point increase in a woman’s PHQ-9 score

Figure: Female Partner's Depressive Symptoms

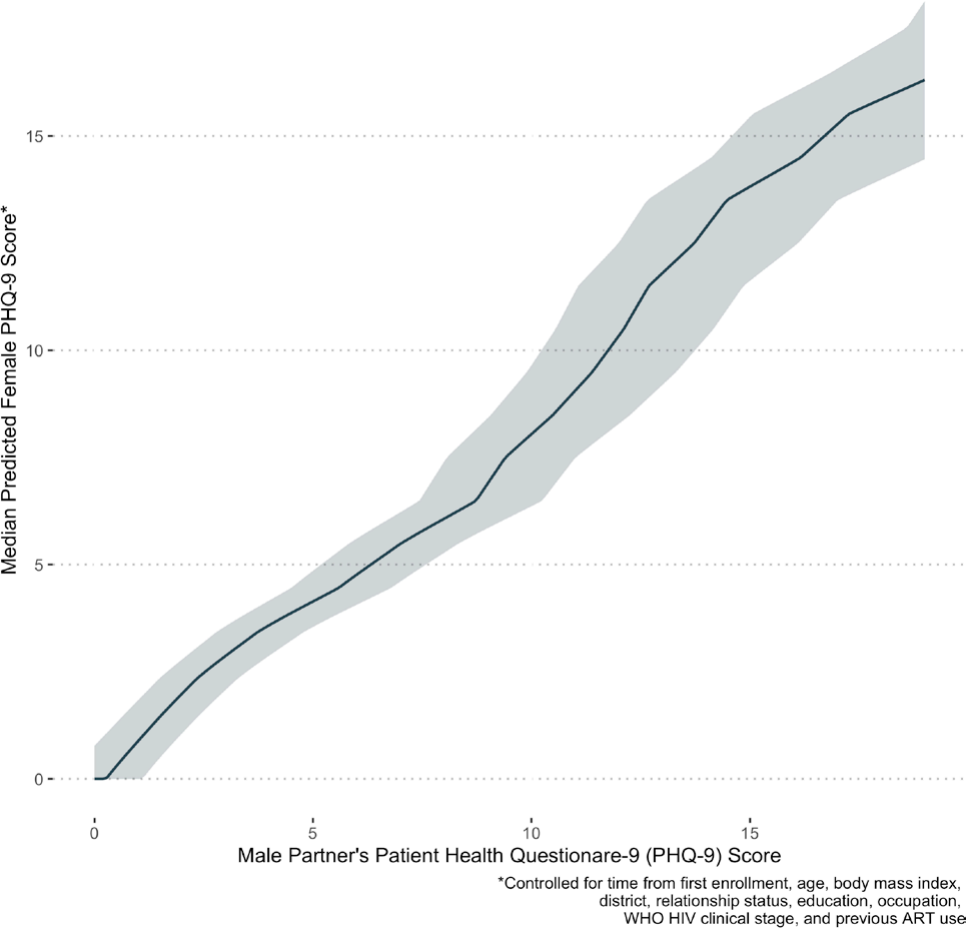

**Conclusion:**

Depressive symptoms are highly correlated among pregnant people and their partners, which may have implications for pregnancy care. Interventions aimed to reduce depressive symptoms and improve HIV-related outcomes during pregnancy may have greater success when focused on addressing both partners’ depressive symptoms.

**Disclosures:**

**All Authors**: No reported disclosures

